# Supplementary material for: The cistrome response to hypoxia in human umbilical vein endothelial cells
Source: eLife. 2026 Aug 6;15:RP111508. doi: 10.7554/eLife.111508 (PMC13446901; doi:10.7554/eLife.111508)
Supplement: Supplementary file 1 [file elife-111508-mdarchecklist1.docx]

**Materials Design Analysis Reporting (MDAR)**

**Checklist for Authors**

The [MDAR framework](https://osf.io/xfpn4/) establishes a minimum set of requirements in transparent reporting mainly applicable to studies in the life sciences.

*eLife* asks authors to **provide detailed information within their article** to facilitate the interpretation and replication of their work. Authors can also upload supporting materials to comply with relevant reporting guidelines for health-related research (see [EQUATOR Network](http://www.equator-network.org/%20)), life science research (see the [BioSharing Information Resource](http://biosharing.org/)), or animal research (see the [ARRIVE Guidelines](http://www.plosbiology.org/article/info:doi/10.1371/journal.pbio.1000412) and the [STRANGE Framework](https://doi.org/10.1038/d41586-020-01751-5); for details, see *eLife*’s [Journal Policies](https://reviewer.elifesciences.org/author-guide/journal-policies)). Where applicable, authors should refer to any relevant reporting standards materials in this form.

For all that apply, please note **where in the article** the information is provided. Please note that we also collect information about data availability and ethics in the submission form.

**Materials:**

| **Newly created materials** | **Indicate where provided: section/figure legend** | **N/A** |
| --- | --- | --- |
| The manuscript includes a dedicated "materials availability statement" providing transparent disclosure about availability of newly created materials including details on how materials can be accessed and describing any restrictions on access. | N/A | N/A |
|  |  |  |
| **Antibodies** | **Indicate where provided: section/figure legend** | **N/A** |
| For commercial reagents, provide supplier name, catalogue number and [RRID](https://scicrunch.org/resources), if available. | N/A | N/A |
|  |  |  |
| **DNA and RNA sequences** | **Indicate where provided: section/figure legend** | **N/A** |
| Short novel DNA or RNA including primers, probes: Sequences should be included or deposited in a public repository. | N/A | N/A |
|  |  |  |
| **Cell materials** | **Indicate where provided: section/figure legend** | **N/A** |
| Cell lines: Provide species information, strain. Provide accession number in repository OR supplier name, catalog number, clone number, OR RRID. | Found in Materials and Methods – MOA-seq library preparation and sequencing section. The study used commercially available human umbilical vein endothelial cells (HUVECs; Homo sapiens; ATCC, Cat. No. PSC-100-010) maintained between passages 5 and 7 under standard culture conditions. Cells were certified as mycoplasma-free by the supplier, and STR authentication was not performed. Control MOA libraries were generated using human A549 lung adenocarcinoma cells (Homo sapiens; ATCC, Cat. No. CCL-185). |  |
| Primary cultures: Provide species, strain, sex of origin, genetic modification status. | HUVECs (ATCC, Cat. No. PSC-100-010) source and information provided in first section of Materials and Methods | N/A f |
|  |  |  |
| **Experimental animals** | **Indicate where provided: section/figure legend** | **N/A** |
| Laboratory animals or Model organisms: Provide species, strain, sex, age, genetic modification status. Provide accession number in repository OR supplier name, catalog number, clone number, OR RRID. | N/A | N/A |
| Animal observed in or captured from the field: Provide species, sex, and age where possible. | N/A | N/A |
|  |  |  |
| **Plants and microbes** | **Indicate where provided: section/figure legend** | **N/A** |
| Plants: provide species and strain, ecotype and cultivar where relevant, unique accession number if available, and source (including location for collected wild specimens). | N/A | N/A |
| Microbes: provide species and strain, unique accession number if available, and source. | N/A | N/A |
|  |  |  |
| **Human research participants** | **Indicate where provided: section/figure legend) or state if these demographics were not collected** | **N/A** |
| If collected and within the bounds of privacy constraints report on age, sex, gender and ethnicity for all study participants. | N/A | N/A |

**Design:**

| **Study protocol** | **Indicate where provided: section/figure legend** | **N/A** |
| --- | --- | --- |
| If the study protocol has been pre-registered, provide DOI. For clinical trials, provide the trial registration number OR cite DOI. | N/A | N/A |
|  |  |  |
| **Laboratory protocol** | **Indicate where provided: section/figure legend** | **N/A** |
| Provide DOI OR other citation details if detailed step-by-step protocols are available. | The laboratory protocol is described in the Materials and Methods – MOA-seq library preparation and sequencing section of the manuscript, which details HUVEC cell culture, hypoxia treatment, nuclei isolation, MNase digestion, DNA extraction, library preparation, quality assessment, and Illumina sequencing. No external step-by-step protocol is available. |  |
|  |  |  |
| **Experimental study design (statistics details) *** | | |
| **For in vivo studies: State whether and how the following have been done** | **Indicate where provided: section/figure legend. If it could have been done, but was not, write “not done”** | **N/A** |
| Sample size determination | N/A | N/A |
| Randomisation | N/A | N/A |
| Blinding | N/A | N/A |
| Inclusion/exclusion criteria | N/A | N/A |
|  |  |  |
| **Sample definition and in-laboratory replication** | **Indicate where provided: section/figure legend** | **N/A** |
| State number of times the experiment was replicated in the laboratory. | Found in Materials and Methods – MOA-seq library preparation and sequencing; Within sample and differential peak calling sections. Two independent biological replicates were performed for each experimental condition (normoxia, 1-hour hypoxia, 3-hour hypoxia, and 24-hour hypoxia). The number of biological replicates was selected based on established practice for genome-wide chromatin profiling experiments. Peak reproducibility between biological replicates was evaluated using the Irreproducible Discovery Rate (IDR) framework in accordance with ENCODE guidelines. No biological replicate samples were excluded as outliers. Reads and peaks were filtered according to predefined quality-control criteria, including mapping quality, PCR duplicate removal, blacklist regions, control regions, and IDR reproducibility. |  |
| Define whether data describe technical or biological replicates. | Found in Materials and Methods – MOA-seq library preparation and sequencing; Within sample and differential peak calling sections. The data presented throughout the study represent biological replicates. Two independent biological replicates were generated for each condition and used for all sequencing and downstream analyses. Technical replicates were not performed or analyzed separately. |  |
|  |  |  |
| **Ethics** | **Indicate where provided: section/submission form** | **N/A** |
| Studies involving human participants: State details of authority granting ethics approval (IRB or equivalent committee(s), provide reference number for approval. | N/A | N/A |
| Studies involving experimental animals: State details of authority granting ethics approval (IRB or equivalent committee(s), provide reference number for approval. | N/A | N/A |
| Studies involving specimen and field samples: State if relevant permits obtained, provide details of authority approving study; if none were required, explain why. | N/A | N/A |
|  |  |  |
| **Dual Use Research of Concern (DURC)** | **Indicate where provided: section/submission form** | **N/A** |
| If study is subject to dual use research of concern regulations, state the authority granting approval and reference number for the regulatory approval. | N/A | N/A |

**Analysis:**

| **Attrition** | **Indicate where provided: section/figure legend** | **N/A** |
| --- | --- | --- |
| Describe whether exclusion criteria were pre-established. Report if sample or data points were omitted from analysis. If yes, report if this was due to attrition or intentional exclusion and provide justification. | In Materials and Methods – Within sample and differential peak calling section. Exclusion criteria were pre-established as part of the bioinformatic analysis pipeline. Reads with a mapping quality score (MAPQ) below 20, PCR duplicates, ENCODE blacklist regions, and MOA control regions were excluded prior to downstream analyses. No biological samples were excluded after sequencing. Peak reproducibility was assessed using the Irreproducible Discovery Rate (IDR) framework, and only reproducible peaks were retained for subsequent analyses. |  |
|  |  |  |
| **Statistics** | **Indicate where provided: section/figure legend** | **N/A** |
| Describe statistical tests used and justify choice of tests. | In Materials and Methods – Within sample and differential peak calling; MOA-seq enrichment analyses; Hierarchical clustering analysis; Motif discovery and analysis; Gene Set Enrichment Analysis sections. Statistical analyses were selected based on the type of genomic data being analyzed. Peak reproducibility between biological replicates was assessed using the Irreproducible Discovery Rate (IDR) framework. Differential transcription factor occupancy was identified using the MACS3 bdgdiff algorithm, with the likelihood ratio cutoff determined by the elbow method. Enrichment analyses were performed using permutation-based z-score calculations (100 randomized iterations), Enrichr, and Gene Set Enrichment Analysis (GSEA) implemented through the clusterProfiler package. Differential gene expression was analyzed using DESeq2 with an adjusted p-value threshold of ≤0.05. Motif enrichment analyses were performed using XSTREME and SEA from the MEME Suite. These methods were selected because they are widely accepted and appropriate for identifying reproducible peaks, differential chromatin occupancy, enriched pathways, differential gene expression, and transcription factor binding motifs in high-throughput sequencing datasets. |  |
|  |  |  |
| **Data availability** | **Indicate where provided: section/submission form** | **N/A** |
| For newly created and reused datasets, the manuscript includes a data availability statement that provides details for access (or notes restrictions on access). | Found in Data Access section. The manuscript includes a Data Availability statement describing the public availability of all newly generated datasets. MOA-seq sequencing data have been deposited in the NCBI Sequence Read Archive (SRA) under accession PRJNA1217882, and processed MOA-seq BigWig and peak files have been deposited in the Gene Expression Omnibus (GEO) under accession GSE289863. The datasets are publicly accessible through these repositories. |  |
| When newly created datasets are publicly available, provide accession number in repository OR DOI and licensing details where available. | Found in Data Access section. All newly generated MOA-seq datasets are publicly available through the NCBI Sequence Read Archive (SRA) under accession PRJNA1217882 and the Gene Expression Omnibus (GEO) under accession GSE289863. These repositories provide open public access to the sequencing data and processed files. |  |
| If reused data is publicly available provide accession number in repository OR DOI, OR URL, OR citation. | The study also reused publicly available datasets, including ENCODE bulk RNA-seq, candidate cis-regulatory element (cCRE), DNase I hypersensitivity, and transcription factor ChIP-seq datasets from the ReMap 2022 database. These datasets are cited in the manuscript through their corresponding publications and accession identifiers where available. |  |
|  |  |  |
| **Code availability** | **Indicate where provided: section/figure legend** | **N/A** |
| For any computer code/software/mathematical algorithms essential for replicating the main findings of the study, whether newly generated or re-used, the manuscript includes a data availability statement that provides details for access or notes restrictions. | Found in Materials and Methods – Gene Set Enrichment Analysis section. The manuscript includes a Code Availability statement describing the availability of all custom code required to reproduce the computational analyses. Custom MOA-seq processing and analysis scripts have been deposited on Zenodo (DOI: 10.5281/zenodo.16595154) and are also publicly available through the GitHub repository (https://github.com/as5906/HypoxiaMOA-HUVEC). No restrictions apply to accessing these resources. |  |
| Where newly generated code is publicly available, provide accession number in repository, OR DOI OR URL and licensing details where available. State any restrictions on code availability or accessibility. | Found in Materials and Methods – Gene Set Enrichment Analysis section. All newly developed analysis scripts used for MOA-seq processing and downstream analyses are publicly available through Zenodo (DOI: 10.5281/zenodo.16595154) and GitHub (https://github.com/as5906/HypoxiaMOA-HUVEC). The code is publicly available through Zenodo and GitHub. The repository contains the scripts required to reproduce the computational analyses presented in this study. |  |
| If reused code is publicly available provide accession number in repository OR DOI OR URL, OR citation. | The study also utilized publicly available software packages, including Cutadapt, FLASh, BWA-MEM2, SAMtools, BEDTools, MACS3, IDR, ChIPseeker, deepTools, the MEME Suite (XSTREME and SEA), DESeq2, clusterProfiler, and Enrichr. These software tools are cited in the Materials and Methods section through their corresponding publications. |  |

**Reporting:**

The MDAR framework recommends adoption of discipline-specific guidelines, established and endorsed through community initiatives.

| **Adherence to community standards** | **Indicate where provided: section/figure legend** | **N/A** |
| --- | --- | --- |
| State if relevant guidelines (e.g., ICMJE, MIBBI, ARRIVE, STRANGE) have been followed, and whether a checklist (e.g., CONSORT, PRISMA, ARRIVE) is provided with the manuscript. | N/A  No discipline-specific reporting guideline (e.g., CONSORT, PRISMA, ARRIVE, or STRANGE) was applicable. The study follows standard reporting practices for genomic sequencing analyses using publicly available software and ENCODE-recommended quality assessment procedures. | N/A |

* We provide the following guidance regarding transparent reporting and statistics; we also refer authors to [Ten common statistical mistakes to watch out for when writing or reviewing a manuscript](https://doi.org/10.7554/eLife.48175).

**Sample-size estimation**

- You should state whether an appropriate sample size was computed when the study was being designed
- You should state the statistical method of sample size computation and any required assumptions
- If no explicit power analysis was used, you should describe how you decided what sample (replicate) size (number) to use

**Replicates**

- You should report how often each experiment was performed
- You should include a definition of biological versus technical replication
- The data obtained should be provided and sufficient information should be provided to indicate the number of independent biological and/or technical replicates
- If you encountered any outliers, you should describe how these were handled
- Criteria for exclusion/inclusion of data should be clearly stated
- High-throughput sequence data should be uploaded before submission, with a private link for reviewers provided (these are available from both GEO and ArrayExpress)

**Statistical reporting**

- Statistical analysis methods should be described and justified
- Raw data should be presented in figures whenever informative to do so (typically when N per group is less than 10)
- For each experiment, you should identify the statistical tests used, exact values of N, definitions of center, methods of multiple test correction, and dispersion and precision measures (e.g., mean, median, SD, SEM, confidence intervals; and, for the major substantive results, a measure of effect size (e.g., Pearson's r, Cohen's d)
- Report exact p-values wherever possible alongside the summary statistics and 95% confidence intervals. These should be reported for all key questions and not only when the p-value is less than 0.05.

**Group allocation**

- Indicate how samples were allocated into experimental groups (in the case of clinical studies, please specify allocation to treatment method); if randomization was used, please also state if restricted randomization was applied
- Indicate if masking was used during group allocation, data collection and/or data analysis
