# Supplementary material for: The cistrome response to hypoxia in human umbilical vein endothelial cells
Source: eLife. 2026 Aug 6;15:RP111508. doi: 10.7554/eLife.111508 (PMC13446901; doi:10.7554/eLife.111508)
Supplement: Supplementary file 2 [file elife-111508-supp12.zip › MEME/cluster1/fimo_out_2/fimo.html]

FIMO Results


---

|  |  |  |  |  |  |
| --- | --- | --- | --- | --- | --- |
| **Database and Motifs** | **High-scoring Motif Occurences** | **Debugging Information** | **Results in TSV Format** | **Results in GFF3 Format** | **Best Site per Sequence** |

  
  


---
