# Supplementary material for: The cistrome response to hypoxia in human umbilical vein endothelial cells
Source: eLife. 2026 Aug 6;15:RP111508. doi: 10.7554/eLife.111508 (PMC13446901; doi:10.7554/eLife.111508)
Supplement: Supplementary file 2 [file elife-111508-supp12.zip › MEME/cluster5/xstreme.html]

XSTREME Results


[close ]

[close ]

[
close ]

[
close ]

[
close ]

[
close ]

[
close ]

[
close ]

[
close ]

[
close ]

[
close ]

[
close ]

# XSTREME

## Motif Discovery and Enrichment Analysis

For further information on how to interpret these results please access
https://meme-suite.org/meme/doc/xstreme-output-format.html.  
To get a copy of the MEME software please access
https://meme-suite.org.

Motifs
  |  
Programs
  |  
Input Files
  |  
Program information
  |  
Summary in TSV Format 
  |  
Non-redundant Motifs in MEME Text Format


# Javascript is required to view these results!

# Your browser does not support canvas!


## Motifs

**Enriched motifs
(E-value ≤ ).**

Expand All Clusters
Collapse All Clusters

## Programs

## Input Files

#### Alphabet


#### Motifs

##### XSTREME version

(Release date: )

##### Command line
