# Supplementary material for: The cistrome response to hypoxia in human umbilical vein endothelial cells
Source: eLife. 2026 Aug 6;15:RP111508. doi: 10.7554/eLife.111508 (PMC13446901; doi:10.7554/eLife.111508)
Supplement: Peer review history

# Editor report: eLife Assessment

**DOI:** 10.7554/eLife.111508.3.sa0

**Authors:** Yamini Dalal

**Affiliations:** National Cancer Institute United States

**Keywords:** Convincing, Important

This is an **important** study that applies a new chromatin profiling technique to the study of cellular responses to low oxygen. The authors provide **convincing** evidence for distinct kinetic phases of the response and identify many new putative regulators of the response. This work will be of broad interest to those studying low oxygen responses and transcriptional regulation.

# Referee report: Reviewer #1 (Public review)

**DOI:** 10.7554/eLife.111508.3.sa1

[Editors' note: this version has been assessed by the Reviewing Editor without further input from the original reviewers. The authors have satisfactorily addressed the comments raised in the previous round of review with textual revisions.]

Summary:

The manuscript by Singh et al. presents an application of MOA-seq to better define transcriptional control underlying the hypoxia response in human endothelial cells. This group's previously described MOA-seq technique allows for precise, identity-agnostic mapping of occupied sites of DNA-binding proteins across the epigenome and over time. Here, they applied MOA-seq to HUVECs under normal oxygen conditions or variable lengths of hypoxia treatment, comparing changes in occupancy over time and associating these changes with corresponding transcriptome alterations. This approach revealed thousands of dynamically occupied sites comprising 10 major kinetic clusters that appear to define distinct subsets and phases of the hypoxia response. Analysis of DNA motifs in these dynamically occupied regions captured the known major roles of HIF1A in the hypoxia response and also implicated new HIF1A-associated regulators. Importantly, they also identified many potential HIF1A-independent candidate TFs that act at HREs, which has been an outstanding question in the field. Additionally, this study identified ~7K additional sites not previously defined as regulatory elements by ENCODE.

Strengths:

Overall, this study is well executed and described, providing new biological insights as well as a rich data resource for the field. As MOA-seq was previously developed for use in plants, this work demonstrates the application of this method in mammalian cells and highlights its utility in identifying new potential regulatory sites not captured by DNase-seq or ATAC-seq. The conclusions made by the authors are well supported by the results, with the caveat that extensive use of DNA motif identification and ontology analyses invariably leads to some uncertainty regarding factor identity and gene network properties.

# Referee report: Reviewer #2 (Public review)

**DOI:** 10.7554/eLife.111508.3.sa2

Summary:

Singh et al. apply MOA-seq to map transcription factor occupancy genome-wide in HUVECs across a hypoxia time course. The study provides a well-validated, high-resolution view of cistrome dynamics and identifies both HIF1A-associated and independent regulatory programs.

Major comments from the first round of review:

Methodological validation is strong. MOA-seq's ability to map protein-bound DNA at near-nucleotide resolution without factor-specific antibodies is a genuine advance, and the cross-validation against independent ChIP-seq and ENCODE datasets is convincing. As noted, future work with additional biological replicates could further strengthen confidence in the smaller kinetic clusters.

Imaging-based validation would strengthen the key biological claims. The kinetic clustering and pathway enrichments are computationally inferred. Orthogonal approaches, for example, live-cell fluorescence imaging of HIF1A nuclear translocation to confirm the proposed temporal binding waves, would provide independent experimental support.

# Author comment: Author response

**DOI:** 10.7554/eLife.111508.3.sa3

**Authors:** Ayush Singh, Viktor Pastukh, Justin T Roberts, Zachary M Turpin, Zehta S Glover, Grant T Daly, Jane M Benoit, Mark N Gillespie, Hank W Bass

**Affiliations:** Florida State University Tallahassee United States; University of South Alabama Mobile United States; University of South Alabama Mobile United States; Florida State University Tallahassee United States; Florida State University Tallahassee United States; University of South Alabama Mobile United States; Florida State University Tallahassee United States; University of South Alabama Mobile United States; Florida State University Tallahassee United States

The following is the authors’ response to the original reviews.

**Public Reviews:**

**Reviewer #1 (Public review):**

Summary:

The manuscript by Singh et al. presents an application of MOA-seq to better define transcriptional control underlying the hypoxia response in human endothelial cells. This group's previously described MOA-seq technique allows for precise, identity-agnostic mapping of occupied sites of DNA-binding proteins across the epigenome and over time. Here, they applied MOA-seq to HUVECs under normal oxygen conditions or variable lengths of hypoxia treatment, comparing changes in occupancy over time and associating these changes with corresponding transcriptome alterations. This approach revealed thousands of dynamically occupied sites comprising 10 major kinetic clusters that appear to define distinct subsets and phases of the hypoxia response. Analysis of DNA motifs in these dynamically occupied regions captured the known major roles of HIF1A in the hypoxia response and also implicated new HIF1A-associated regulators. Importantly, they also identified many potential HIF1A-independent candidate TFs that act at HREs, which has been an outstanding question in the field. Additionally, this study identified ~7K additional sites not previously defined as regulatory elements by ENCODE.

Strengths:

Overall, this study is well executed and described, providing new biological insights as well as a rich data resource for the field. As MOA-seq was previously developed for use in plants, this work demonstrates the application of this method in mammalian cells and highlights its utility in identifying new potential regulatory sites not captured by DNase-seq or ATAC-seq. The conclusions made by the authors are well supported by the results, with the caveat that extensive use of DNA motif identification and ontology analyses invariably leads to some uncertainty regarding factor identity and gene network properties.

Weaknesses:

There are several areas where the clarity of presentation could be improved:

(1) Given the importance of the methodology, the methods section needs more detail on how the extent of MNase digestion is chosen to achieve optimal results with MOA-seq. This is described to some extent in the description of control library preparation, but not for the experimental samples.

We thank the reviewer for noting this unintended omission. We have not updated the Methods section to specify as follows:

"Digestion patterns were assessed via gel electrophoresis, and the light digest levels ideal for MOA-seq (as per Savadel et al., 2021) were selected as the lightest digest levels that give a pattern of a nucleosomal ladder spanning the entire DNA fragment size range from undigested to mononucleosome bands, as indicated in Figure 1 with the asterisk-marked gel lanes."

(2) The abstract describes this approach as "native cistrome profiling" but this is misleading since formaldehyde fixation is used.

We believe the formaldehyde fixation captures native chromatin structure, but indeed we are digesting fixed chromatin and have updated the wording to read as “in situ cistrome profiling.”

(3) Species- and field-specific jargon and abbreviations need to be clarified on first usage. For example, on page 9: "Downsampling analysis was carried out for two sets of published reference peaks; the CTCF cCRE peak midpoints and for the ERG motif under the ERG ReMap ChIP-seq peaks." The different categories of cCREs were not clearly defined, nor will it be clear what the term ReMap refers to for those outside the field. The sentence after this refers to IDR, which also should be defined.

We thank the reviewer for highlighting the need for clearer definitions of field-specific terminology and abbreviations. In response, we have revised the manuscript to explicitly define all relevant terms at first mention. Specifically, we now describe the ENCODE candidate cis-regulatory element (cCRE) catalogue and define the individual cCRE categories, including promoter-like (PLS), proximal enhancer-like (pELS), distal enhancer-like (dELS), DNase I–H3K4me3 (K4m3), and CTCF-only regions. We also clarify that ReMap is a curated database of human transcriptional regulator binding peaks derived from ChIP-seq, ChIP-exo, and DAP-seq experiments. Additionally, we now define IDR as the Irreproducible Discovery Rate framework upon first use.

(4) Figure 4C: Are these motifs examined under MOA sites specifically or anywhere in the genes in question?

Leading up to and including Figure 4C, we have not yet examined any motifs. Instead, Figure 4C compares gene sets, one defined by our diff-MOA, and those from GO libraries, in this case the "target genes" which are defined by TF-specific studies, primarily ChIP-seq but also related immuno-based mapping techniques. Consequently, the analysis shown in Fig. 4C is not a motif enrichment analysis. Instead, we used the ENRICHR gene set enrichment analysis tool with ENCODE and ChEA consensus transcription factor target gene sets. Thus, the analysis was performed at the gene-set level, and transcription factor motifs were not examined within diff-MOA peaks or elsewhere in the associated genes for Fig. 4C. We note that motif enrichment within diff-MOA peaks was subsequently examined separately in Fig. 6. In Fig. 7, we further examined differentially expressed genes associated with diff-MOA peaks containing enriched transcription factor motifs and used clustering analyses to investigate their regulatory relationships. We have clarified these distinctions in the revised manuscript.

If the question is about the location of MOA footprints relative to gene structure, we did not examine any MOA sites at any specific location, just overlapping the gene +/- 200 bp, as indicated in Fig. 4B.

(5) Figure 5B shows that up-DEGs with diff-MOA footprints tend to show more losses of footprints. Do the authors interpret this as a loss of repressor binding?

Not exclusively, but yes, that is one plausible explanation. That is, the activation (defined by increased RNA levels) via de-repression could be happening. But we also expect these dynamic footprints to be but one component. In other words, we interpret the relationship as consistent with that possibility, but not only that possibility. A logical explanation is that loss of footprint occupancy associated with upregulated genes could be based on displacement of repressive DNA-binding factors, thereby contributing to transcriptional activation. Thus, while loss of repressor binding is a plausible explanation for a subset of these events, additional factor-specific experiments would be required to know for sure in each case. We have added text to the Discussion acknowledging this possibility.

**Reviewer #2 (Public review):**

Summary:

Singh et al. apply MOA-seq to map transcription factor occupancy genome-wide in HUVECs across a hypoxia time course. The study provides a well-validated, high-resolution view of cistrome dynamics and identifies both HIF1A-associated and independent regulatory programs.

Major Comments:

Methodological validation is strong. MOA-seq's ability to map protein-bound DNA at near-nucleotide resolution without factor-specific antibodies is a genuine advance, and the cross-validation against independent ChIP-seq and ENCODE datasets is convincing. As noted, future work with additional biological replicates could further strengthen confidence in the smaller kinetic clusters.

Regarding additional biological replicates, we have acknowledged this point in the discussion. Importantly, we did subject the replicates to IDR analysis, which we explain in the methods as "In accordance with ENCODE ChIP-seq guidelines (Landt et al., 2012), we further evaluated data quality by assessing pooled pseudo-replicate consistency and self-consistency for each individual replicate (Supplementary Table S2)." This IDR analysis demonstrated consistent peaks between our bioreplicates, meeting ENCODE guidelines. In addition, downsampling analysis demonstrated that our sequencing depth of coverage (Supp Fig 1) was over 10-fold greater than required. We do appreciate that it will be useful to have more biological replicates from other cell types, tissues, or organisms, and hope this study prompts just such future research.

Imaging-based validation would strengthen the key biological claims. The kinetic clustering and pathway enrichments are computationally inferred. Orthogonal approaches, for example, live-cell fluorescence imaging of HIF1A nuclear translocation to confirm the proposed temporal binding waves, would provide independent experimental support.

Live-cell imaging could indeed be interesting, but it is beyond our current capacity to add to this study and consider this an exciting future direction, but presence in the nucleus could include both bound and unbound HIF1, so the results may not easily track the DNA-bound HIF1 only.

**Recommendations for the authors:**

**Reviewer #1 (Recommendations for the authors):**

In Figure 3B, the x-axis is not labeled.

Thank you for pointing this out. We have revised Figure 3B by adding the previously missing x-axis label.

**Reviewer #2 (Recommendations for the authors):**

In the abstract, it would be good to define what MOA-seq is and what the cistrome is.

Thank you for this suggestion. We have revised the abstract to define both MOA-seq (MNase-defined cistrome-Occupancy Analysis sequencing) and the cistrome upon first mention to improve accessibility for readers who may be unfamiliar with these terms.
